# Advances in HIV-1 Envelope-Based Vaccine Development

**DOI:** 10.3390/vaccines14100914

**Published:** 2026-10-21

**Authors:** Kun-Wei Chan, Xunqing Jiang, Andrew L. Wang, Xiang-Peng Kong, Xueling Wu

**Affiliations:** 1 Department of Medicine, Section of Infectious Diseases, Boston Medical Center, Chobanian & Avedisian School of Medicine, Boston University, Boston, MA 02118, USA; kun-wei.chan@bmc.org; 2 Department of Biochemistry and Molecular Pharmacology, New York University Grossman School of Medicine, New York, NY 10016, USA; xunqing.jiang@nyulangone.org; 3 Undergraduate Program in Biology, Boston University, Boston, MA 02215, USA; andrewlw@bu.edu

**Keywords:** HIV-1, envelope, vaccine, broadly neutralizing antibody, germline targeting

## Abstract

As the sole viral protein expressed on the surface of viral particles and infected cells, the HIV-1 envelope glycoprotein is the most important target for vaccine development. Structurally defined sites of vulnerability recognized by broadly neutralizing antibodies (bNAbs) serve as major targets for vaccine design, including V1V2 apex, V3 base glycan, CD4-binding site, fusion peptide, and membrane proximal external region. Although well-established for affinity-matured antibody binding and antigenicity, these sites are not necessarily immunogenic. Three approaches have been undertaken to work around this problem: stabilizing native-like trimers, targeting bNAb germlines, and developing booster immunizations based on envelope-bNAb coevolution. With these approaches, breakthroughs have been made in vaccine-elicited neutralization gaining breadth, thus supporting certain immunogens and regimens over others. Here, from the perspectives of HIV-1 envelope and antibody genetics as well as structures, we systematically review the most recent advances in envelope-based vaccines and their remaining barriers. We also summarize lessons learned about HIV-1 envelope immunogenicity and discuss potential strategies for future development.

## 1. Introduction

Belonging to the family of Retroviridae, HIV-1 infects susceptible host cells—primarily CD4+ T cells—through the cellular receptor CD4 and co-receptor CCR5 (or CXCR4 to a lesser extent). Upon infection, HIV-1 integrates its reverse transcribed viral DNA into the human genome to establish a provirus and viral reservoir that cannot be cleared with the current standard of care. Hence, lifelong adherence to antiretroviral therapy (ART) is required for viral control. Likewise, ART-based pre-exposure prophylaxis (PrEP) also requires consistent adherence to be effective, posing a significant challenge for HIV-1 care and prevention. To alleviate the burden for daily oral PrEP, two long-acting injectables, Apretude (cabotegravir) every other month and Yeztugo (lenacapavir) twice yearly, were approved for HIV-1 PrEP in 2021 and 2025, respectively. However, adherence is still a requirement, and its associated time commitment and high cost also restrict uptake, with long-acting injectables accounting for only 5% or less in all active PrEP prescriptions. Hence, an effective vaccine is likely required to end the epidemic.

**Figure 1:**
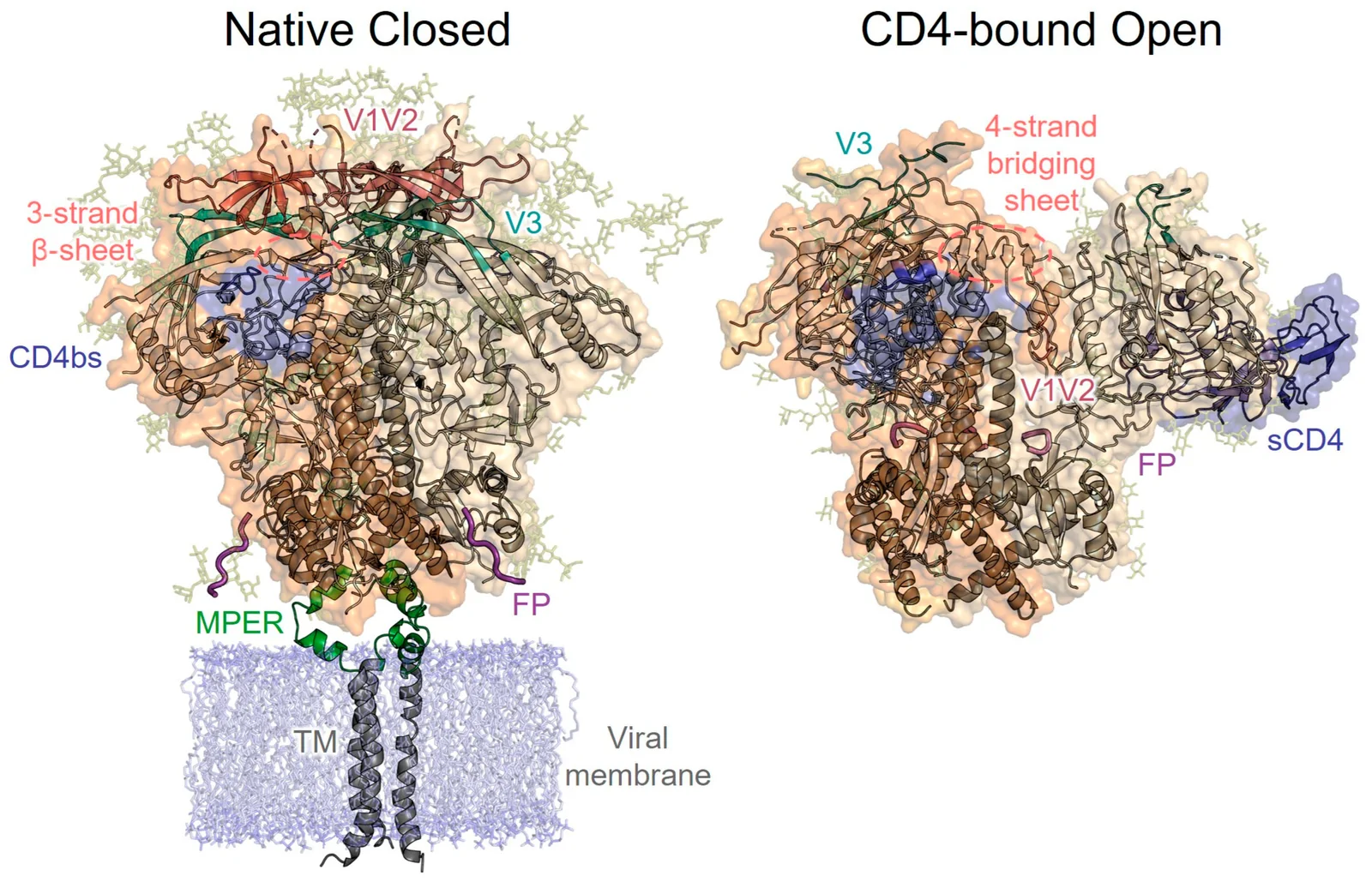
Structural overview of HIV-1 Env trimer and key sites of vulnerability. The HIV-1 Env trimer model is based on the SOSIP ectodomain (PDB 5FYL) and MPER-TM (6DLN), shown as ribbons in the native closed conformation (**left**). The ectodomain is also shown as semi-transparent surface, with each protomer colored in distinct shades of orange and glycans displayed as sticks. Key sites of vulnerability are highlighted: V1V2 (dark pink), V3 (teal), CD4bs (blue patch), FP (purple), and MPER (dark green). The CD4-bound open conformation (5VN3, (**right**)) illustrates the displacement of V1V2, exposure of V3, and rearrangement of the three-strand β-sheet into a four-strand bridging sheet (reddish dashed circles) following soluble CD4 (sCD4) binding. The membrane bilayer was modeled using CHARMM-GUI Membrane Builder [11]. All figures were prepared using PyMOL v3.1.4.1 (Schrödinger, LLC, New York, NY, USA).

As the only viral protein expressed on the surface of viral particles and infected cells, the HIV-1 envelope (Env) glycoprotein is the most important vaccine target to elicit antibodies that neutralize the virus. Over a hundred broadly neutralizing antibodies (bNAbs) have been isolated from HIV-1-infected individuals, often from chronic infections. As HIV-1 is effective at evolving antibody-escape mechanisms, bNAbs generated after established infections are merely products of Env-antibody coevolution that remain a step behind viral mutations. To be protective, bNAbs need to be present at the site of viral exposure before infection, resonating with the goal of eliciting bNAb responses from prophylactic vaccines. The bNAb epitopes have been structurally mapped to nearly the entire exposed surface of native Env, revealing multiple sites of vulnerability (Figure 1, left), including V1V2 apex, V3 base glycan, and CD4-binding site (CD4bs) on the surface unit gp120, as well as fusion peptide (FP) and membrane proximal external region (MPER) of the transmembrane (TM) unit gp41. Packaged into a trimer, HIV-1 Env mediates viral entry through a series of conformational changes. For example, binding to the CD4 receptor triggers V1V2 displacement and opening of V3 at the trimer apex to form the co-receptor-binding site (Figure 1, right), and the engagement of a co-receptor, primarily CCR5, leads to further changes in gp41 to expose the FP and mediate membrane fusion. This Env metastable organization makes it challenging to present the native closed trimer recognized by bNAbs, contributing to the limited effectiveness of early Env immunogens. Hence, when native-like trimer designs were developed, such as the fully cleaved SOSIP (disulfide bond linking gp120-gp41 and Ile/Pro mutation in gp41) in 2013 [1] and the cleavage-independent NFL (native flexibly linked) in 2015 [2], they raised high hopes for effective Env trimer immunogens. However, subsequent production and immunization studies showed that their respective prototype BG505 SOSIP and JR-FL NFL trimers—albeit still among the most widely used Env immunogens in the field—were ineffective at inducing bNAbs. One notable issue associated with the SOSIP trimers was the presence of “glycan holes,” which are off-target and highly strain-specific [3,4]. Simple glycan knock-in [5] did not help, which may be attributed to the overall poor immunogenicity of HIV-1 Env [6]. Another problem was the presence of non-native neo-epitopes at the base of soluble trimers [4,7], which was later partially addressed by membrane-bound [8,9] and liposome-arrayed Env trimers [10].

Genetic features of HIV-1 bNAbs have also indicated two major obstacles: the rarity of germline precursors in the human B-cell receptor (BCR) repertoire and improbable somatic hypermutations (SHMs) required for antibody affinity maturation. Both issues appear more profound for HIV-1 Env as compared with other viral glycoproteins, likely due to an especially dense glycan shield of HIV-1 Env and its greater genetic diversity accumulated through chronic infections. To address the first obstacle, the field has designed germline-targeting (GT) immunogens that preferentially select rare bNAb precursors based on increased binding to inferred bNAb germline or unmutated common ancestor (UCA). This approach requires single-cell sequencing of post-immune BCR repertoires, in addition to conventional post-immune assessment of polyclonal serum or plasma. BCR sequencing showed that some GT immunogens enriched bNAb precursors as compared with naïve, unvaccinated controls in non-human primates (NHPs) and humans. However, these efforts have not yet overcome the second obstacle—inducing improbable SHMs required for bNAb affinity maturation—which has now become the most pressing challenge. The field hypothesizes that after appropriate priming (i.e., activation of bNAb germlines or UCAs), multiple rounds of booster immunizations can guide the SHM and affinity maturation required for bNAb functions. To test this hypothesis, one promising approach is sequential immunizations based on Env-antibody coevolution, which the field has started to experiment with.

Here we systematically review the most recent advances in Env immunogens and immunization trials pertaining to each site of vulnerability of HIV-1 Env, from the perspectives of HIV-1 Env and antibody genetics as well as structures. We also offer our perspective on future Env immunization strategies.

## 2. HIV-1 Env Immunogens Targeting the V1V2 Apex

The V1V2 apex is a major target of HIV-1 bNAbs [12,13]. The prototype V1V2-directed bNAbs, PG9 and PG16, first reported in 2009, neutralized 70–80% of tested viruses [14]. Subsequent bNAb lineages, including PGT141–145 [12] and their distal clonal members PGDM1400–1412 [15], and later CH01–04 [16] and CAP256–VRC26 [17], revealed that bNAbs to this site can achieve exceptional potency, with 50% inhibitory concentration (IC_50_) values as low as 0.003 µg/mL. Notably, V1V2 bNAbs are detected relatively early during HIV-1 infection [17,18] and generally exhibit only moderate levels of SHM (~15–25%) compared to other bNAb classes [19], further supporting the V1V2 apex as a vaccine target. However, most V1V2 bNAbs rely on unusually long (24–37 amino acids by Kabat numbering) heavy-chain complementarity-determining region 3 (CDR-H3) loops for Env recognition, suggesting that compatible precursors in the human naïve BCR repertoire may be relatively rare, thereby posing a challenge for germline priming.

**Figure 2:**
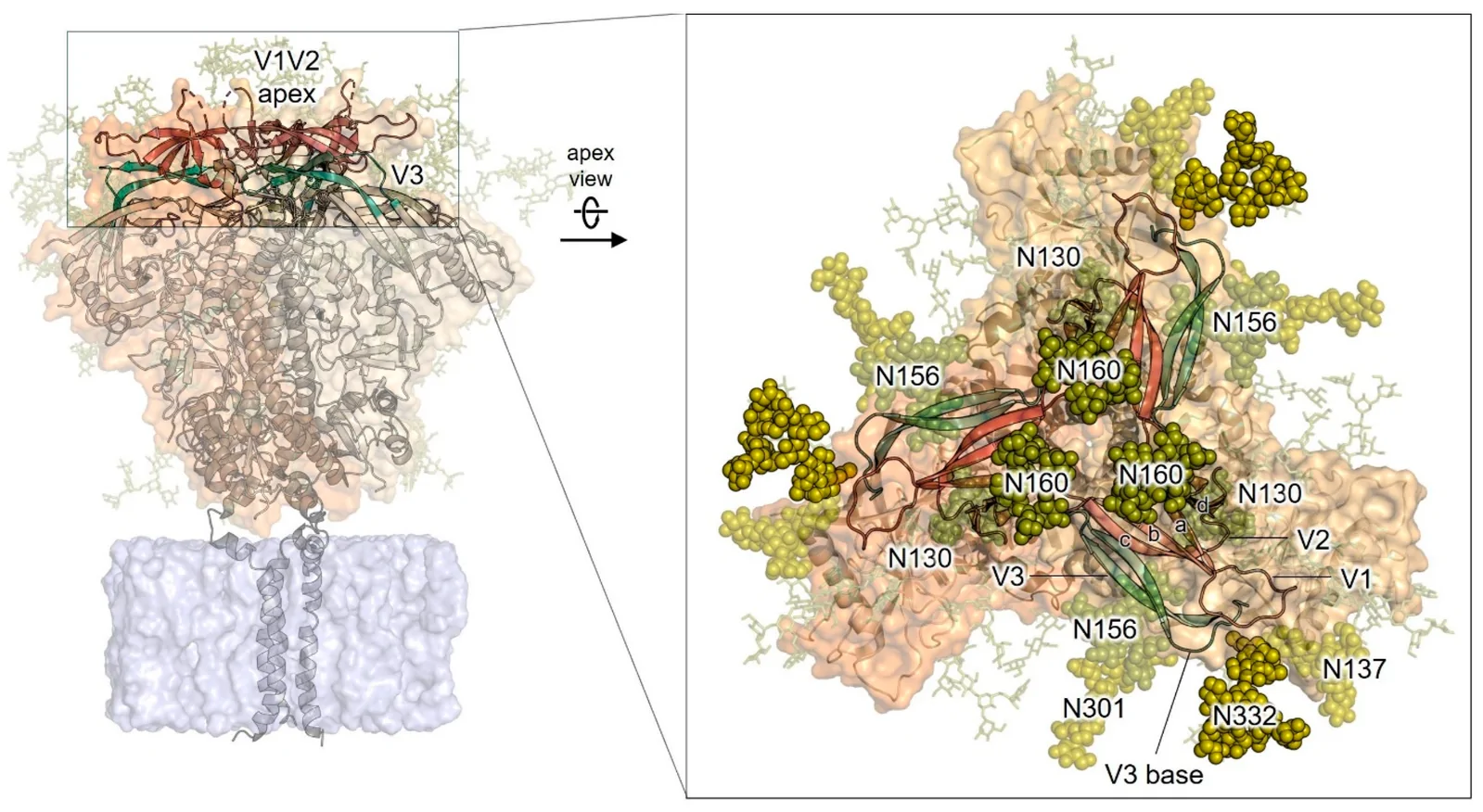
Sites of vulnerability at the Env trimer apex. A side view of the Env trimer is shown with a corresponding close-up in apex orientation, revealing the threefold arrangement of V1V2 and V3 across the protomers. The structural elements of V1V2 (dark pink) and its key N160 glycan (olive spheres) are highlighted, along with other surrounding glycans at N130 and N156. The V3 base (teal) is shown with its key N332 glycan, along with other surrounding glycans at N137 and N301. The V3 base is partially occluded by the V1 loop in the native Env trimer.

On the native Env trimer, V1V2 is a quaternary, glycan-dependent structure formed at the interface of the three gp120 protomers at the trimer apex [20] (Figure 2). V1V2 bNAbs typically employ long, acidic CDR-H3 loops that project toward the trimer axis, penetrating between the V2 N160 glycan and adjacent glycans to engage conserved basic residues such as K169 on strand C of V2 [21,22,23]. The V1V2 region is intrinsically dynamic and undergoes substantial rearrangement upon CD4 binding [24], leading to the exposure of V3 and formation of the co-receptor binding site. As V1V2 bNAbs recognize Env in its native closed conformation, it is likely necessary to present the quaternary V1V2 apex in the context of a native-like closed trimer, posing another challenge for immunogen design. Indeed, prior designs have attempted to present V1V2 on scaffolds [25], which elicited limited neutralizing antibody (nAb) responses, reflecting the difficulty of recapitulating key features of V1V2 in its native closed trimer form.

Given the strict trimer dependence of V1V2 bNAbs, immunogen development has focused on stabilized native-like trimers. The BG505 (clade A) Env was selected for native-like SOSIP trimer construction based on its similarity to Env sequences in the individual from whom the V1V2 bNAbs PG9 and PG16 were isolated [26]. Major modifications to create BG505 SOSIP included the removal of gp41 TM and cytoplasmic tail to create a soluble gp140, A501C and T605C substitutions to form a disulfide bond between gp120 and gp41_ECTO_, I559P substitution to promote trimerization, replacement of the native cleavage site with an optimal sequence (RRRRRR; R6) for full cleavage, truncation of MPER from residue-664 to prevent aggregation, and T332N substitution to restore a bNAb-dependent N332 glycan [1]. BG505 SOSIP and its related trimers elicited autologous nAbs in animal models [27,28] but with limited responses against the apex region [29]. Early clinical studies also demonstrated autologous nAb responses. In the Phase I HIV Vaccine Trials Network (HVTN) 137A trial (NCT04177355), soluble BG505 SOSIP formulated with adjuvant 3M-052-AF in Alum induced nAbs in 5 of 15 vaccine recipients, with some targeting the trimer apex; nevertheless, heterologous neutralization was not detected [30]. Another Env, Q23 (clade A), was selected as a priming immunogen based on its binding to the rhesus apex-targeting bNAb RHA1 [31] and its inferred germline, followed by ZM233 (clade C) and four additional Envs as booster immunogens [32]. Confirmed by monoclonal antibodies (mAbs), liposomes displaying NFL trimers from these Envs, along with adjuvant SMNP (saponin QS-21 with monophosphoryl lipid A nanoparticle), elicited apex-directed heterologous bNAbs in NHPs, with the broadest mAb achieving >50% neutralization breadth against a panel of non-clade B viruses lacking the N130 glycan. Although these vaccine-induced antibodies structurally resembled bNAbs PG9 and CH03, their neutralization was limited by the presence of N130 glycan. To further understand the barriers to eliciting V1V2 bNAbs, a study of 122 simian-human immunodeficiency virus (SHIV)-infected NHPs identified 5 Envs that preferentially elicited V1V2 bNAbs in 18 animals [33]. Env-antibody coevolution revealed that B-cell priming, rather than subsequent affinity maturation, was the principal bottleneck to V1V2 bNAb development.

Given the importance of B-cell priming, GT was implemented to engage rare V1V2 bNAb precursors with long CDR-H3. Analysis of 14 human BCR repertoires identified potential precursors for two V1V2 bNAbs, PCT64 and PG9, and ApexGT trimers were designed to bind the inferred germlines of these bNAbs [34]. Both soluble and membrane-bound ApexGT5 were tested in PCT64 germline knock-in mice and elicited antibodies to V2 but showed no neutralization [35]. A further optimized trimer, ApexGT6, was evaluated in NHPs and shown to activate B-cell precursors with CDR-H3 features of multiple V1V2 bNAbs, but without heterologous neutralization [36]. Another set of GT immunogens—CAP256.OPT4 and OPT4-S—derived from the Env CAP256SU (clade C), elicited V1V2 bNAbs in 5 of 5 macaques, with two neutralizing > 50% of a 15-virus panel and one of them > 70% [37]. In a subsequent lineage-based regimen, booster immunogens incorporating the N130 glycan and coevolution-identified Env variants elicited bNAbs in 8 of 8 macaques, extending neutralization to N130 glycan-containing viruses. Whether these advances can be translated into humans remains to be seen.

## 3. HIV-1 Env Immunogens Targeting the V3 Base Glycan Supersite

The HIV-1 Env is protected by a dense glycan shield, helping the virus evade antibodies. However, some antibodies can exploit specific glycan clusters that are highly conserved on HIV-1 Env. More specifically, glycans surrounding the base of the V3 loop have been identified as particularly vulnerable to antibody-mediated neutralization (Figure 2). The first breakthrough regarding this vulnerability came in 2011 when crystal structures showed that bNAbs PGT127 and PGT128 could penetrate the glycan shield and engage two conserved mannose-rich glycans together with the β-strand at the V3 base [38]. The N332 glycosylation site was subsequently identified as a particularly conserved target recognized by multiple bNAbs, including PGT121, 10-1074, PGT128, PGT135, and BG18, establishing the N332-centered region at the V3 base as a supersite of vulnerability [39].

The N332-targeting bNAbs feature potent antiviral neutralization (geometric mean IC_50_s around 0.02 µg/mL), long CDR-H3s (18–24 amino acids), and moderately high SHMs (17–20%). Although the N332 supersite is one of the most promising leads for immunogen design, the wild-type Env trimer did not readily activate these bNAb precursors. To solve this issue, additional mutations were introduced into BG505 SOSIP to increase binding affinity for PGT121 UCA, leading to MD39-based GT trimer designs [40]. The subsequent N332-GT5 trimer was designed to target BG18-like precursors [41] and successfully primed such precursors in NHPs [42]. Two booster immunogens, B11 and B16, were evaluated in a BG18 precursor-transfer mouse model and further drove SHM [43]. However, in contrast to the prototype bNAb BG18, antibodies from these immunizations targeted V1 but not N332 of the booster strain. As shown in Figure 2, the proximity of V1 can obscure the N332 supersite at the V3 base. Four additional booster immunogens, B46, B50, B54, and B20, along with other Envs, guided N332-GT5-primed antibody lineages in NHPs to achieve up to two-thirds of the neutralization breadth observed for the N332-directed reference bNAbs [44].

A recent SHIV infection study provided new insights into this target. In macaques infected with SHIV.5MUT (modified from SHIV.BG505.N332), early V1-directed antibodies selected Env variants with shortened and less glycosylated V1 loops, which subsequently primed bNAb precursors to the N332 supersite [45]. Similarly, an N332-targeting bNAb DH1030 was isolated from a SHIV.BG505.N332-infected macaque, and the inferred UCA of DH1030 neutralized an autologous Env with a shortened V1 as compared with the infecting SHIV.BG505.N332 [46], thus supporting the use of shortened-V1 Envs as priming immunogens.

## 4. HIV-1 Env Immunogens Targeting the CD4-Binding Site

**Figure 3:**
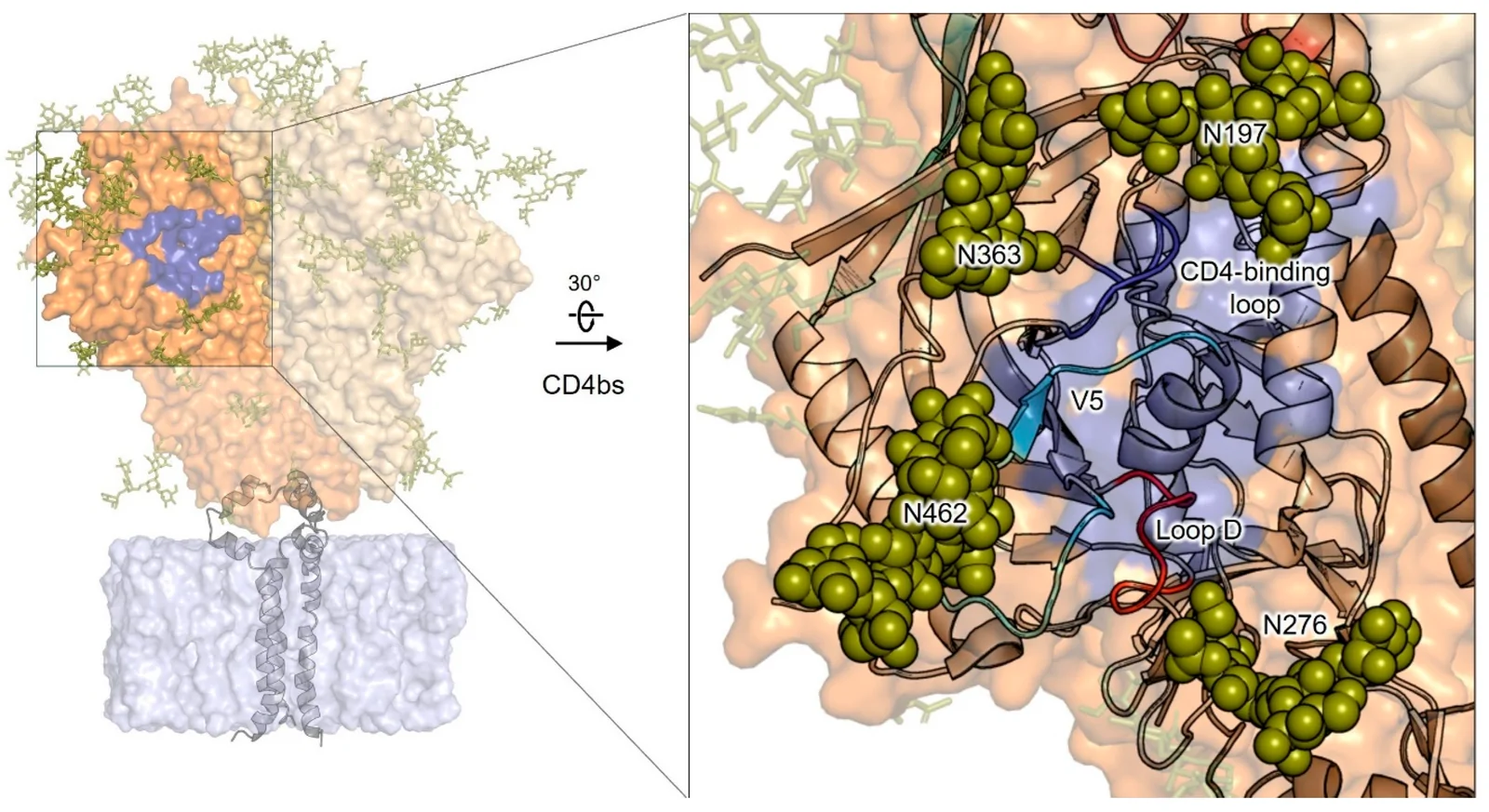
Close-up view of the CD4-binding site (CD4bs). The HIV-1 Env trimer is shown with the CD4bs indicated by a blue surface patch, together with a ribbon close-up view. Key elements of the CD4bs are indicated, including the CD4-binding loop (blue), V5 loop (cyan), and Loop D (red), with the surrounding glycans at N197, N276, N363, and N462 displayed as olive spheres.

Given their favorable neutralization breadth (>90%) and potency (geometric mean IC_50_s around 0.2 µg/mL), bNAbs targeting the CD4bs of gp120 have long been the focus of Env immunogen design. In this regard, the CD4bs has several favorable properties: it is immunogenic, well conserved, and exposed on the outer domain (OD) of gp120 core, facilitating the production of domain-based immunogens. However, the CD4bs also presents two challenges: gp120 monomers act as decoys by presenting non-neutralizing epitopes such as the inner domains and variable loops, especially V1 and V3, which are also immunodominant; furthermore, there are surrounding glycans that protect the CD4bs (Figure 3). Hence, in addition to compatibility with the closed trimer, specific binding modes and angles are also required for bNAbs to avoid or accommodate the surrounding glycans to reach their protein epitopes. For these reasons, prior immunogens were able to induce antibody responses to the CD4bs; however, these antibodies were non-neutralizing, effective only against tier-1 viruses, or weakly neutralizing against a small fraction of tier-2 viruses [47], due to incompatibility with closed trimers or occlusion by surrounding glycans.

Since its discovery, VRC01 and its class of CD4bs bNAbs [48,49,50,51,52] have served as the prototype for GT immunogen design. The VRC01-class bNAbs share three genetic features: IGHV1-2, high levels of SHM (25–35%), and a short, five-amino-acid CDR-L3 [48]. A variety of cell lines, mouse models, and recombinant proteins were developed to model or display the IGHV1-2 germline and inferred VRC01 or 3BNC117 UCAs, enabling the selection of immunogens based on their ability to bind and activate the bNAb UCAs. Among the immunogens tested, a series of engineered outer domain germline-targeting (eOD-GT) 60-mers were the most advanced. In a phase I clinical trial (IAVI G001), two intramuscular injections of eOD-GT8 60-mer adjuvanted with AS01_B_ induced VRC01-class bNAb precursors in 97% (35 of 36) of vaccine recipients, as compared with 17% (2 of 12) of placebo [53]. Although comparison with vaccine recipients from prior trials that showed no efficacy would provide a more stringent benchmark, the near-universal detection of VRC01-class precursors in vaccine recipients established the efficient priming by eOD-GT8 60-mer. However, the eOD-GT8 60-mer-induced VRC01-class precursors lacked detectable affinity for native Env trimers or neutralization.

An alternative approach is to induce the CD4bs bNAbs, e.g., CH103, that neutralize the virus via CDR-H3 and require less SHM [54]. In the HVTN 300 trial, a CH505 (clade C) Env chimeric trimer induced antibodies suggestive of CD4bs bNAb precursors in 4 of 11 participants [55]. However, the antibody specificity in polyclonal sera was still primarily directed to the off-target trimer base, lacking heterologous tier-2 neutralization. Hence, a major challenge here is to develop booster immunogens that can guide further affinity maturation toward functional CD4bs bNAbs.

## 5. HIV-1 Env Immunogens Targeting the Fusion Peptide

The HIV-1 FP is a 16–20 amino acid segment located at the N-terminus of the Env TM unit gp41. This hydrophobic segment functions as a molecular harpoon during viral entry: following the engagement with receptor and co-receptor, FP inserts into the host cell membrane to initiate membrane fusion, a step essential for viral infection [56,57,58]. Because this function is indispensable, FP is under strong evolutionary constraint and is highly conserved across global HIV-1 isolates. Known bNAbs such as VRC34.01 [59], ACS202 [60], and PGT151 [61] can target this epitope, establishing FP as a site of vulnerability for vaccine design.

**Figure 4:**
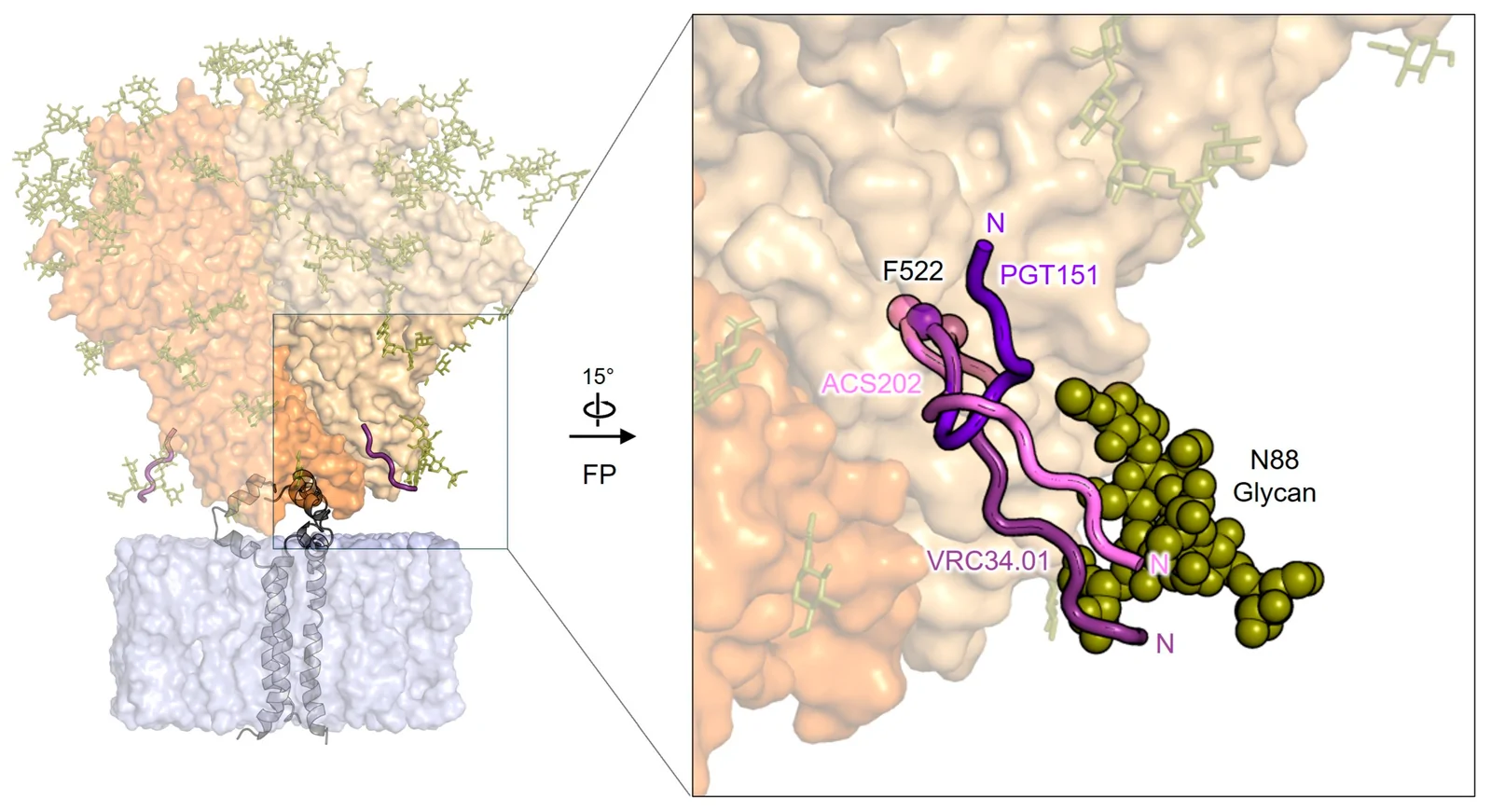
Close-up view of the fusion peptide (FP) region. FP conformations from different bNAb-bound structures (PDB 6NC3 for bNAb VRC34.01, 8TOX for bNAb ACS202, 6OLP for bNAb PGT151) are aligned onto the Env trimer. Only the N-terminal segment (residues 512–522) is shown, with F522 displayed as spheres, highlighting differences in FP conformation and orientation in the Env trimer context. The indicated N88 glycan is a key component of the FP-targeting bNAb epitopes.

The N-terminal eight residues of FP (FP8), with ^512^AVGIGAVF^519^ being the most common among global HIV-1 isolates, form the primary epitope recognized by antibodies [62]. Structural studies demonstrated distinct modes of bNAb recognition of FP (Figure 4). For example, bNAb VRC34.01 recognizes a composite epitope comprising the N-terminal free FP8 and the conserved N88 glycan on gp120, highlighting the importance of Env cleavage for this epitope presentation [59,63,64]. On the other hand, bNAb ACS202 stabilizes FP in an extended β-strand that forms an antiparallel β-sheet with its CDR-H3 loop [60,64], whereas PGT151 recognizes a broader quaternary epitope at the gp120–gp41 interface that incorporates FP in a different orientation [61]. Structural analyses also identified F522 as a pivot residue that enables rotation of the peptide, allowing antibodies to recognize the peptide from multiple angles [65]. Although the FP N-terminus is partially exposed in the native closed trimer, FP is intrinsically flexible, reflecting its role in Env conformational rearrangements required for membrane fusion, thus presenting a challenge for stable epitope presentation in Env immunogens.

Immunization with BG505 SOSIP and related Env trimers elicited FP-specific antibodies with weak and narrow neutralizing activity in NHPs [4,66,67] and humans (VRC 018 trial) [68]. Structural analyses indicated that antibody lineages, such as rhesus RM20F [67] and human N751-2C06 and N751-2C09 [68], interacted with the FP as well as the C1 conserved N88 glycan. As the non-native neo-epitopes at the base of SOSIP can account for up to 90% of vaccine-elicited antibody response [69,70], an epitope-based prime–boost strategy was applied to focus the antibody response toward FP. FP8 peptides conjugated to carrier proteins—such as keyhole limpet hemocyanin (KLH) [62,69], recombinant tetanus toxoid heavy chain (rTTHc) [71], or virus-like particles (VLPs) [72]—initiate FP-specific B-cell lineages. Subsequent boosting with stabilized native-like Env trimers, including BG505 DS-SOSIP, refines antibody specificity and enhances affinity for the native Env [62,72]. Macaques primed with FP and then challenged with SHIV developed neutralization breadths of 45–77% within weeks, driven by rapid affinity maturation of FP-primed antibody lineages [73].

To prevent repeated off-target stimulations during sequential immunizations, glycans have been added to mask shared carrier epitopes [72]. To mimic the prolonged antigen exposure during HIV-1 infection, implantable osmotic pumps delivering immunogens continuously over several months extended germinal center activity and promoted additional rounds of B-cell maturation in NHPs [74]. More recently, dual-epitope SOSIPs targeting both FP and V1V2 apex generated higher autologous neutralization titers in NHPs while also improving cross-strain neutralization breadth [75,76]. Despite these efforts, it remains particularly challenging to identify booster immunogens that can guide FP-directed antibody maturation.

## 6. HIV-1 Env Immunogens Targeting the Membrane Proximal External Region

The gp41 MPER is a highly conserved segment at the base of native Env that is targeted by some of the broadest bNAbs described to date [77], yet it remains a challenging target for vaccine development [78]. Early MPER-directed bNAbs, such as 2F5 and 4E10, first defined this region as a site of vulnerability [79,80]. Later identification of the more potent bNAb 10E8, capable of neutralizing ~98% of tested viruses [77], further underscored the potential of MPER as a vaccine target. MPER-directed bNAbs often recognize both peptide and lipid components and feature relatively extended CDR-H3s enriched in hydrophobic and aromatic residues that enable membrane-proximal engagement [81], suggesting their development may be constrained by immune tolerance [82,83].

**Figure 5:**
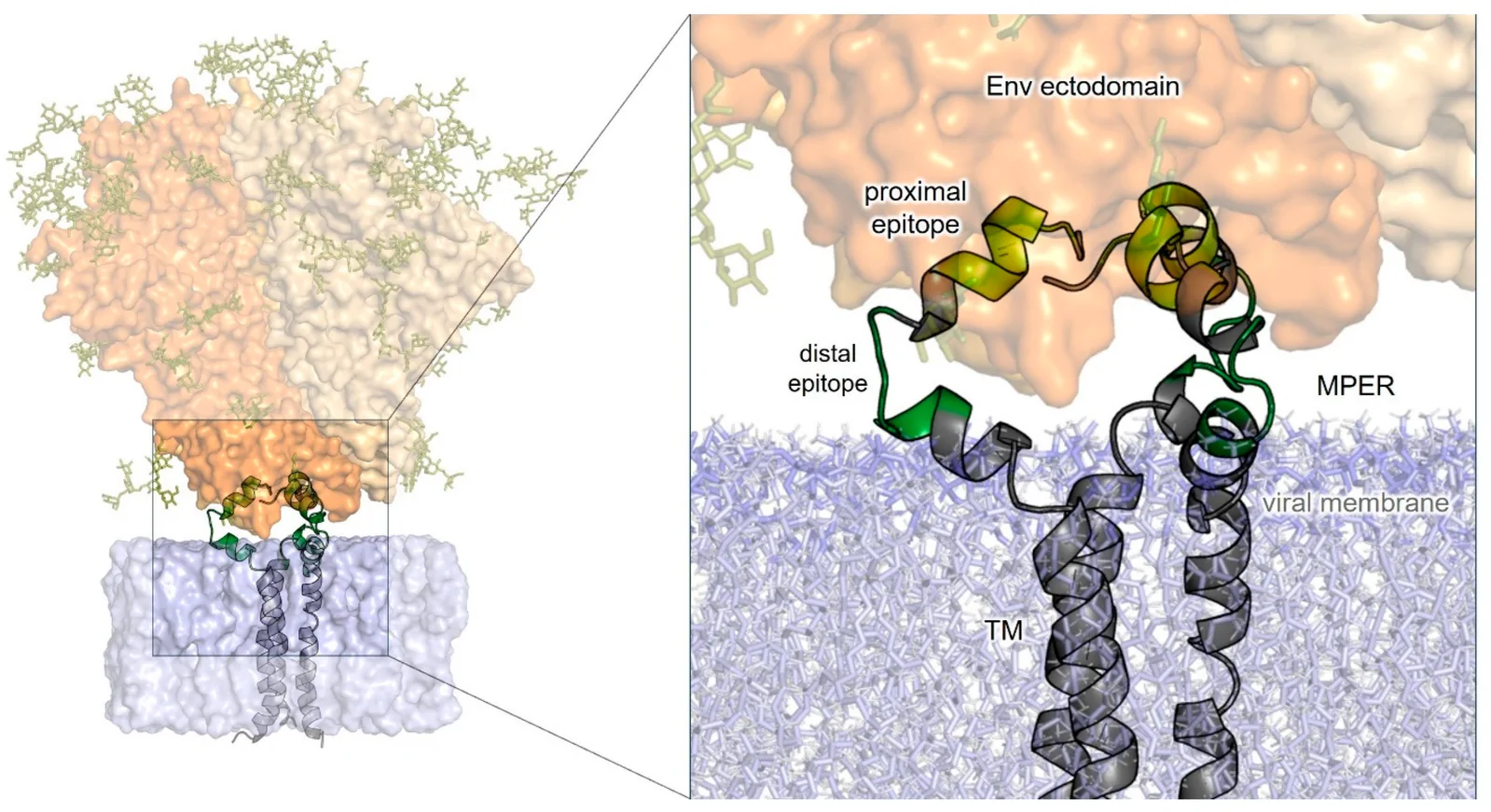
Close-up view of the MPER region at the base of Env trimer and the membrane interface. Based on bNAb epitopes, the MPER can be divided into a proximal region (light green, residues 662–667, adjacent to the ectodomain) and a distal region (dark green, residues 671–676, positioned along the membrane surface). As shown, the MPER is largely occluded by the ectodomain, limiting its epitope accessibility.

Structurally, MPER comprises a short segment immediately N-terminal to the TM domain and lies at the membrane interface [84,85]. Based on the epitopes targeted by bNAbs, this region is typically divided into proximal (targeted by 2F5) and distal (targeted by 4E10 and 10E8) epitopes relative to the ectodomain of Env (Figure 5). In membrane-mimetic environments, MPER adopts helical conformations that can extend from the gp41 ectodomain into the TM region [84]. Neutralization by MPER bNAbs is therefore closely linked to membrane proximity and epitope accessibility, with antibody-lipid interactions facilitating engagement of partially occluded peptide determinants [78,86].

These structural and immunological features collectively pose two central challenges for immunogen design. First, the MPER resides at the Env base, partially shielded by the membrane and trimer architecture [86]. To improve solubility, many native-like Env trimers truncate gp41 prior to MPER, thereby excluding this site [1]. Second, MPER bNAbs often exhibit lipid-reactivity and autoreactivity, suggesting that immune tolerance may limit precursor availability and affinity maturation [78,87]. Accordingly, earlier MPER epitope-scaffold immunogens lacking an appropriate membrane context elicited binding antibody responses, but no neutralizing activity [88].

Recent clinical evaluation of MPER vaccination has focused on epitope-directed immunogens incorporating a lipid context. In the HVTN 133 trial (NCT03934541), an MPER peptide-liposome elicited serum antibodies and memory B-cell responses against the proximal MPER. Weak and narrow tier-2 neutralization was detected following affinity purification of MPER-specific IgG in a subset of vaccine recipients, although the trial was terminated early because of polyethylene glycol-associated anaphylaxis [89,90]. BCR repertoire analyses demonstrated polyclonal proximal MPER^+^ lineage expansion and acquisition of several improbable mutations associated with gp41 and lipid interactions. Among these, the most potent vaccine-derived human mAb, DH1317.4, is directed to the proximal MPER and mediates weak heterologous neutralization of ~15% of globally representative strains [89]. Together, these findings indicate that MPER peptide-liposome immunogens can initiate bNAb-relevant lineages targeting the proximal MPER in humans, although the breadth and potency remain modest.

Beyond peptide-liposomes, MPER epitope scaffolds established proof-of-concept for structure-guided presentation of the otherwise poorly exposed MPER epitope. T117v2, which lacks a lipid component, bound mature 10E8 but failed to engage any 10E8-class precursors [91,92]. Subsequent optimization of the T117-based MPER scaffolds yielded the 10E8-GT series, among which 10E8-GT10.2 engaged ~60% of 10E8-class precursors, whereas 10E8-GT12 preserved binding to mature 10E8 while maintaining ~46% precursor engagement [92]. Multivalent display on nanoparticles activated 10E8-class precursors in rhesus macaques and stringent mouse models, selecting B-cells with defined CDR-H3 motifs and directional affinity maturation toward more native-like MPER antigens. Delivery by mRNA-lipid nanoparticles elicited comparable precursor responses in mice, supporting platform flexibility [92]. Structural analyses further defined the molecular basis for the enhanced 10E8 affinity of 10E8-GT12 relative to earlier 10E8-GTs, guiding subsequent immunogen refinement [93]. Although demonstrating efficient precursor engagement, current 10E8-GTs have not yet been shown to induce antibodies with mature 10E8-like breadth. Consistent with the overall bNAb GT paradigm, boosting with increasingly native-like MPER or membrane-bound Env immunogens will likely be required to advance B-cell lineage maturation toward broadly neutralizing activity.

## 7. HIV-1 Env Immunogen Summary and Comparison

Even though the binding sites of HIV-1 bNAbs have been mapped to multiple surfaces of the native Env trimer (antigenicity), immunization with a native-like Env trimer does not necessarily induce the same bNAb responses (immunogenicity). As antigenicity measures the antigen binding sites by affinity-matured antibodies, and immunogenicity measures the entire B-cell response, antigenicity does not directly translate into immunogenicity. Hence, native Env trimers are necessary but not sufficient to induce bNAbs. Summarized in Table 1, both unique and common challenges are presented for HIV-1 Env immunogens, targeting different sites of vulnerability. Briefly, a common challenge for those targeting gp120 (V1V2 apex, V3 base glycan, CD4bs) is the need for both priming and boosting native-like trimers involving glycans. The current availability of various critical native-like SOSIP and NFL glycosylated trimers appeared to meet this need and thus advanced immunogens to induce heterologous nAbs gaining breadth by targeting the V1V2 apex and V3 base glycan. However, broad heterologous neutralization targeting the CD4bs has not yet been achieved, likely due to the uniquely required high levels of SHM for this site. In contrast, a shared challenge for those targeting gp41 appears to be the limited accessibility of epitopes (FP and MPER) in native trimers. Therefore, careful considerations given to address the presentation, stabilization, and exposure of these epitopes may benefit their accessibility and overall immunogenicity.

## 8. Discussion

With the advent of native-like SOSIP and NFL trimers, the “original antigenic sin” and immunodominant non-neutralizing epitopes on HIV-1 Env appeared to be less concerning. Instead, the dense glycans that hinder the presentation of B- and T-cell epitopes have become a recurring theme as described above. The “glycan shield” is well established as a mechanism of antibody evasion; conversely, bNAbs have been consistently shown to avoid or interact with glycans for viral recognition. Because HIV-1 Env immunogens must account for viral genetic diversity and membrane-bound trimeric structure, they will inevitably need to incorporate glycans to induce antibodies capable of accommodating or directly interacting with glycans. Viewed as “self,” glycans alone are poorly immunogenic, but this is not necessarily the case for glycoproteins. In fact, a number of immunogen-induced antibodies are reactive with both peptides and glycans; examples include multiple V1V2 apex-directed rhesus mAbs interacting with the N160 glycan [32], multiple V3 base-directed rhesus mAbs interacting with the N332 glycan [44], and FP-directed rhesus mAb RM20F [67] and human mAbs N751-2C06 and N751-2C09 [68] interacting with the C1 conserved N88 glycan.

With the GT approach, immunogens capable of priming rare bNAb precursors have been developed to target multiple sites of vulnerability. However, another recurring theme is the lack of boosting immunogens to guide critical antibody affinity maturation or SHM. Although limited, Env-antibody coevolution studies have started to identify certain Env variants and mutations associated with various bNAb initiation and maturation [33,45,46,95]. Importantly, some of these findings have been applied to immunization tests, including the bNAb lineage-based sequential immunizations that elicited the best V1V2 apex bNAbs in NHPs to date [37]. These advances provide new opportunities to select priming and boosting Envs based on bNAb ontogeny [17,54,96] rather than empirical testing alone. These results also support the hypothesis that after appropriate priming, multiple rounds of booster immunizations can guide the SHM and affinity maturation required for bNAb functions. Further investigations of sequential Env immunizations [37,97] will be critical to determine whether such approaches can reproducibly guide on-target antibody maturation toward greater neutralization breadth and potency.

Although suboptimal, early signs of “on-target” antibody responses have been observed with liposomes displaying native-like trimers, FP, and MPER peptides, often with adjuvants. Multivalent antigens increase vaccine immunogenicity through efficient cross-linking of BCRs and improved antigen trafficking, endocytic uptake, and presentation [98,99]. Therefore, multivalent, arrayed display on liposomes using one or more Env sequences may promote the development of breadth. Using an Env antigen with defined valencies, Kato et al. found that high-valency (60-mer) antigens activated B-cells with a broad range of affinities, and low-valency (4-mer) induced smaller effector B-cell populations that preferentially bind with high affinity [100]. As initial B-cell activation correlates with the magnitude of effector B-cell responses, high-valency is likely to offer the best priming for GT or epitope-focused vaccine designs, but lower-valencies may be better suited to limiting off-target responses if the on-target B-cells bind with high affinity. We also note the importance of on-target response by HIV-1 vaccines and the need to avoid the induction of potentially harmful autoreactive and catalytic antibodies, which have been commonly demonstrated in HIV-1-infected individuals [101]. Additionally, among the adjuvants tested in human trials, AS01_B_ appeared to be superior to MF59 and Alum [102], and SMNP was shown to outperform AS01_B_ and ISCOMs in mice [103]. SMNP was also effective in NHPs [32,103,104] and has been tested in a human trial (HVTN 144, NCT06033209), representing one of the most promising adjuvants tested to date. Despite these advances, vaccine-elicited nAbs often exhibit narrow-to-moderate breadth, leaving them ineffective against other genetically diverse HIV-1 strains and vulnerable to viral escape.

## 9. Conclusions and Perspectives

In conclusion, the overall immunogenicity of HIV-1 Env is unusually weak compared with that of other viral glycoproteins, yet HIV-1 bNAbs are unusually highly mutated compared with other antiviral nAbs. The combined viral and host barriers render the development of HIV-1 Env vaccines exceptionally challenging. Nevertheless, advances in native-like Env trimers, bNAb ontogeny, GT immunogens, multivalent antigen display on liposomes, and adjuvant development have led to early successes in inducing bNAb responses to the V1V2 apex, V3 base glycan, FP, and MPER, supporting further HIV-1 Env immunogen designs and investigations. HIV-1 Env remains a valid vaccine target because of its functional necessity and accessibility at the membrane surface, which together define a persistent vulnerability. Future vaccine strategies will likely focus on sequential immunizations with diverse Env variants and structurally optimized Env trimers designed to guide antibody maturation pathways toward the breadth and durability characteristic of bNAbs.

## Figures and Tables

**Table 1:** HIV-1 Env immunogens summary and comparison.

Target	bNAb Features	Immunogenicity	Immunogen Challenges	Vaccine Approaches	Clinical Trials	Vaccine-Induced Antibodies
V1V2 apex (gp120)	Long CDR-H3	Strong	Quaternary trimer-dependent; Glycan-dependent & glycan-penetrating; Conformationally dynamic	Stabilized trimers; Liposome-displaying trimers; GT trimers; Env-nAb coevolution trimers	Phase I HVTN 137A, NCT04177355 [30]	Human autologous nAbs; NHP > 50% breadth with or without N130 glycan
V3 base glycan (gp120)	Long CDR-H3	Weak	Glycan-dependent; Glycan-penetrating; V1 loop occlusion	GT trimers; Shorter & less glycosylated V1		NHP 2/3 breadth of reference bNAbs
CD4bs (gp120)	High SHM	Strong	Glycan-penetrating; Approaching angle-constrained	Polyvalent gp120; eOD-GT8 60-mer; GT trimers	Phase I DP6-001 [94]; Phase I IAVI G001, NCT03547245 [53]; Phase I HVTN 300, NCT04915768 [55]	Human tier-1 nAbs; Activation of bNAb precursors in humans
FP (gp41)	Moderate SHM	Weak	Small; Hydrophobic; Structurally flexible; Limited accessibility	BG505 DS-SOSIP; Carrier protein-displaying FP8; VLP-displaying FP8	Phase I VRC 018, NCT03783130 [68]	Human and NHP autologous nAbs
MPER (gp41)	Long CDR-H3 Lipid-reactive Autoreactive	Weak	Small; Hydrophobic; Limited accessibility; Lipid component	MPER-liposome; 10E8-GT scaffold	Phase I HVTN 133, NCT03934541 [89]	Human weak heterologous nAbs

## Data Availability

Data is available in the published references.

## Abbreviations

The following abbreviations are used in this manuscript:

HIV-1	Human immunodeficiency virus type 1
Env	Envelope
bNAb	Broadly neutralizing antibody
ART	Antiretroviral therapy
PrEP	Pre-exposure prophylaxis
CD4bs	CD4-binding site
gp120	Glycoprotein 120
FP	Fusion peptide
MPER	Membrane proximal external region
TM	Transmembrane
gp41	Glycoprotein 41
SOSIP	Disulfide bond linking gp120-gp41 and Ile/Pro at gp41 residue 559
NFL	Native flexibly linked
PDB	Protein data bank
BCR	B-cell receptor
SHM	Somatic hypermutation
GT	Germline-targeting
UCA	Unmutated common ancestor
NHP	Non-human primate
IC_50_	50% inhibitory concentration
CDR-H3	Heavy-chain complementarity-determining region 3
nAb	Neutralizing antibody
HVTN	HIV Vaccine Trials Network
NCT	National Clinical Trial
Alum	Aluminum hydroxide gel adjuvant
SMNP	Saponin QS-21 with monophosphoryl lipid A nanoparticle
mAb	Monoclonal antibody
SHIV	Simian-human immunodeficiency virus
OD	Outer domain
IGHV1-2	Immunoglobulin heavy chain variable gene 1-2
CDR-L3	Light-chain complementarity-determining region 3
eOD-GT8	Engineered outer domain-germline targeting 8
IAVI	International AIDS Vaccine Initiative
AS01_B_	Liposome-based adjuvant combining saponin QS-21 with monophosphoryl lipid A
KLH	Keyhole limpet hemocyanin
rTTHc	Recombinant tetanus toxoid heavy chain
VLP	Virus-like particle
MF59	Oil-in-water emulsion adjuvant using squalene
ISCOM	Immune stimulating complexes
